# Properties of an attention-grabbing motion signal: a comparison of tail and body movements in a lizard

**DOI:** 10.1007/s00359-022-01544-3

**Published:** 2022-02-03

**Authors:** Richard A. Peters, Jose Ramos

**Affiliations:** Animal Behaviour Group, Department of Ecology, Environment and Evolution, La Trobe University, Bundoora, VIC 3086 Australia

**Keywords:** Motion signal, Visual amplitude, Plant motion, 3D reconstruction, Lizard

## Abstract

Animals signals must be detected by receiver sensory systems, and overcome a variety of local ecological factors that could otherwise affect their transmission and reception. Habitat structure, competition, avoidance of unintended receivers and varying environmental conditions have all been shown to influence how animals signal. Environmental noise is also crucial, and animals modify their behavior in response to it. Animals generating movement-based visual signals have to contend with wind-blown plants that generate motion noise and can affect the detection of salient movements. The lizard *Amphibolurus muricatus* uses tail flicking at the start of displays to attract attention, and we hypothesized that tail movements are ideally suited to this function. We compared visual amplitudes generated by tail movements with push-ups, which are a key component of the rest of the display. We show that tail movement amplitudes are highly variable over the course of the display but consistently greater than amplitudes generated by push-ups and not constrained by viewing position. We suggest that these features, combined with the tail being a light structure that does not compromise other activities, provide an ideal introductory component for attracting attention in the ecological setting in which they are generated.

## Introduction

Animals exchange signals to influence the behavior of recipients. Paramount to effective signaling is the need to capture attention, and so the sensory and brain properties of receivers mediate whether signals are detected and subsequently processed (Bradbury and Vehrencamp 2011). Indeed, theory suggests that one pathway for signal evolution is to exploit a bias in the sensory capabilities of receivers that is used for other functional tasks (Endler 1992). Even for signals that have evolved via other pathways, it is necessary for the signal structure to match the sensory capabilities of receivers. However, variation in the structure within and between taxa points to a role for other factors contributing to signal structure and signaling behavior. In addition to variations inherent to signaler identity, motivation and context, there are a variety of ecological factors that are major contributors to signal diversity.

Ecological influences on signal structure are many and varied. They include habitat structure (Hunter and Krebs 1979; Slabbekoorn and Smith 2002), competition from other signaling species (Greenfield 1988; Bloomfield et al. 2008), unintended receivers (Ryan et al. 1982; Stoddard 1999, 2002; Steinberg et al. 2014) and the orientation of signalers relative to the sun (Endler 1990; Klomp et al. 2017; Simpson and McGraw 2018a, b). Another key ecological factor affecting the signal structure and signaling behavior is environmental noise, which represents stimulation in the same sensory channel as the signal (Brumm 2014). Importantly, the noise environment is not static and many species experience a change in the noise landscape that requires them to adjust their signals accordingly. The masking effect of noise and resulting adjustments by signalers has received considerable attention in acoustic signaling species, having been reported in diverse taxonomic groups including primates (Brumm et al. 2004), mammals (Parks et al. 2011), birds (Brumm and Todt 2002), reptiles (Brumm and Zollinger 2017) and insects (Römer 2013). Less well known is the masking effect of noise for electrical (Benda et al. 2013) and chemical (Nehring et al. 2013) signals. Visual signals are also subject to noise interfering with reliable detection and processing, including growing appreciation of the role of irrelevant plant motion on the detection of movement-based visual displays (Fleishman 1986; Peters 2008; Bian et al. 2018, 2019) and adjustments made by signalers to overcome environmental motion noise (Ord et al. 2007; Peters et al. 2007; Ord and Stamps 2008).

The behavior of signaling animals in response to noise implies that there are some signal types that are more suited to certain ecological conditions than other signal types, or at other times. A strategy for overcoming adverse signaling conditions is to insert alerting components that serves to attract the attention of receivers. The use of introductory syllables by rufous-sided towhees (*Pipilo erythrophthalmus*) aid in attracting attention and carry little information for receivers (Richards 1981); the introductory notes of many birds may serve a similar function (Kalra et al. 2021). The facultative addition of rapid movements at the start of displays by *Anolis gundlachi* (Ord and Stamps 2008) provides a visual counterpart to the more widely known acoustic signaling feature. Environmental circumstances, therefore, represent a key determining factor in dictating the evolution of signal form.

Many lizard species utilize dynamic visual signals during social interactions (Ramos and Peters 2016), and must deal with the constraints imposed by their signaling environment. Wind-blown plants are the primary source of motion noise for signals defined by movement (Peters 2013) and inhibit the detection of signals by receivers (Peters 2008). Lizards adjust their signaling strategy to offset the masking effects of plant motion in species-specific ways. *Anolis cristatellus* increase display speed (Ord et al. 2007), *A. gundlachi* insert faster, more rapid movements during adverse signaling conditions (Ord and Stamps 2008), while *Amphibolurus muricatus* extend the duration of introductory tail flicking (Peters et al. 2007) before other motor patterns are produced in rapid succession (Peters and Ord 2003). *Amphibolurus muricatus* is an Australian agamid lizard and one of several that have tail movements in their signaling repertoire. However, very few of these species perform tail movements as reliably as *A. muricatus*.

Tail flicking by *A. muricatus* is reported to serve an alerting function that attracts the attention of receivers to the other motor patterns (Peters and Evans 2003). However, it remains unclear why this species relies on tail movements more than other species in the family. We speculate that it has something to do with the interaction of sensory systems and ecology. The habitat utilized by *A. muricatus* throughout much of their geographic distribution is densely vegetated, and they are typically found in close proximity to these plants. They are a semi-arboreal species that will bask where there is adequate sun, either close to the ground or as high as a few meters above ground. When rivals enter their field of view, they respond from their basking position and so their orientation relative to intruders is highly variable. We suggest that the tail is well-suited to sustained movement because it is a light structure that does not compromise the lizard’s ability to respond to other events (e.g. action from the intruder, predators). Another non-mutually exclusive hypothesis is that the movement of tails by *A. muricatus* provides a more optimal signal for attracting attention than alternative movements in their repertoire. The signaler is surrounded by plants, often in close proximity, and the position of the signaler relative to the receiver will be highly variable and across three dimensions of space.

**Fig. 1:**
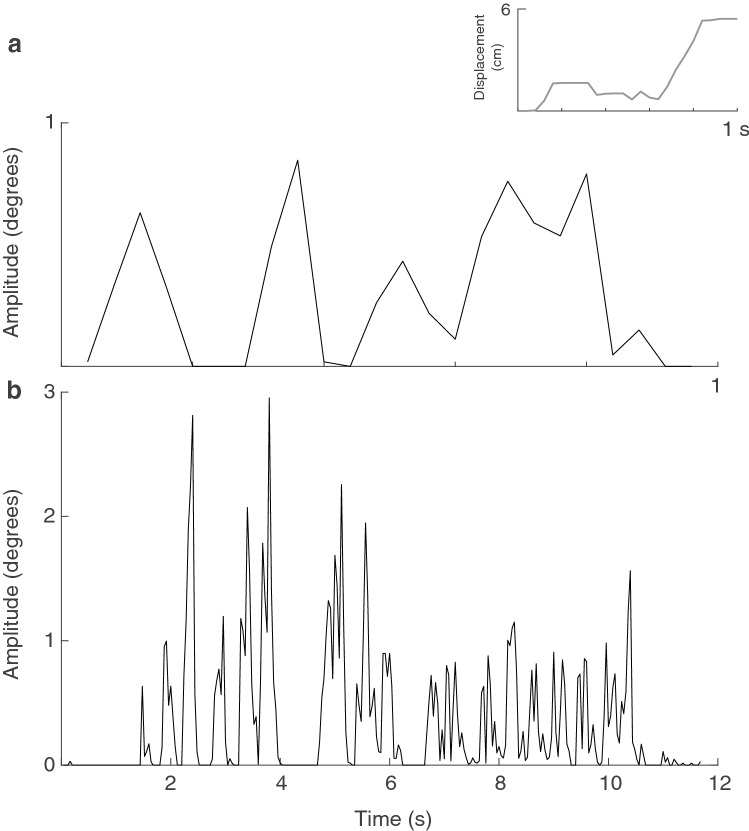
Amplitudes generated by push-ups and tail flicking. **a** The push-up movement was characterized by the movement of the eye. Inset: display action pattern graph showing displacement of the eye over time reveals small movements before a single push up at the end of the short sequence. The main figure depicts visual amplitude between successive frames (25 frames per second) from a viewing distance of 1 m. **b** Tail movements characterized by movement at the tip of the tail between successive frames from a viewing distance of 1 m for a sequence lasting 12 s

Our aim in the present study was to compare the visual amplitude of movements during tail-flicking with that of an alternative movement in their repertoire (Fig. 1). The dominant feature of the second half of the display are push-ups. This motor pattern is relatively common in Australian agamid lizards (Ramos and Peters 2016), as well as species worldwide, and features raising of the head and upper part of the body by movement of the forelegs. Given the propensity for movements to be viewed from above, below and either side of the signaller, we compared amplitudes for both types of movement from the viewpoint of a receiver located anywhere around the lizard. Our general approach was introduced by New and Peters (2010), and used archival footage of wild, free-roaming lizards. We predicted that tail-movements would result in greater amplitudes than push-ups.

## Methods

### Data collection

We used footage of wild *A. muricatus* signaling obtained for a previous study (Ramos and Peters 2017). This study compared two populations of *A. muricatus* from different habitat types: Croajingolong National Park, in coastal Victoria (S37° 48.126′, E149° 16.541′) and the less densely vegetated Avisford Nature Reserve, New South Wales (S32° 37.543′, E149° 33.810′). Full details about the differences between these populations and the filming of the displays are available in the original paper. Briefly, two video cameras (25 frames/s frame rate) were used to film displays of wild, unmarked male lizards in response to a tethered intruder. Recording of a calibration object featuring 20 points at different depths and heights immediately afterwards permitted subsequent computation of calibration coefficients that can be used for three-dimensional (3D) reconstruction of movements (Hedrick 2008; Peters et al. 2016). We selected eight sequences for further analysis, comprising four from each of the two populations.

### Signal analysis

**Fig. 2:**
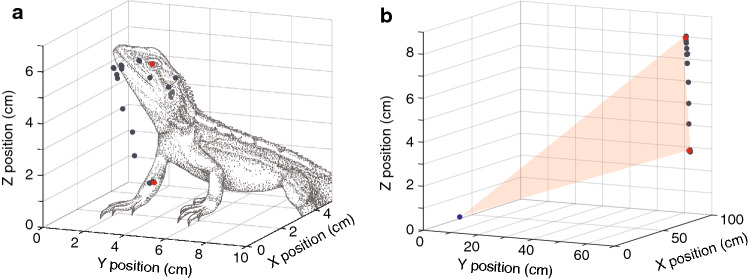
**a** The position of the chosen feature (eye) was tracked in video frames from two video cameras. The separate 2D representations were subsequently combined to represent the position of the feature in 3D space. **b** The visual angle swept by a display movement. The visual angle swept by a display movement (two red dots) at the eye of a receiver (blue dot) is computed, and repeated for all pairwise combinations of points

**Fig. 3:**
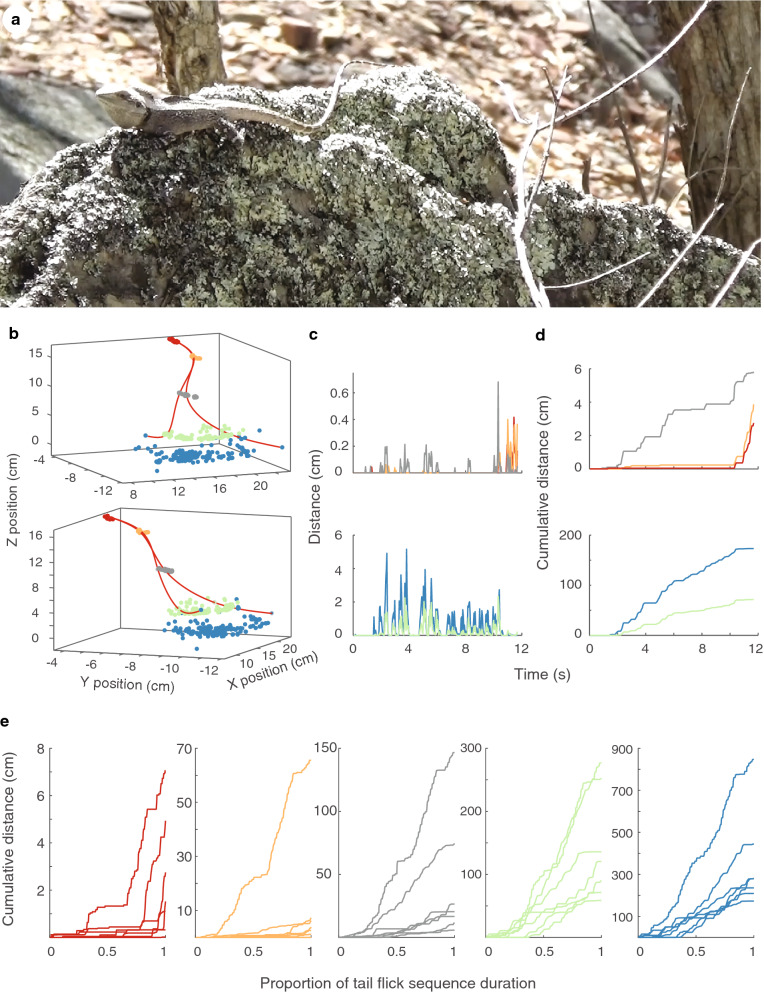
**a** *Amphibolurus muricatus* midway through a tail flicking display. **b** The position of five parts of the tail across the tail flicking display in 3D space, starting at the base of the tail (red) to the tail tip (blue). The red lines represent splines through the five points of a given frame. The two viewing angles illustrate how viewing angle can influence perceived displacement. **c** Distance travelled between successive frames and **d** cumulative distance travelled across the whole display for the base of the tail and next two points (top panel) and the tail tip and nearest point (bottom panel). **e** Cumulative distance travelled as a function of relative sequence duration for eight different displays. Different plots represent different parts of the tail from the base of the tail (red) to the tail tip (blue). Note the different scales on the y-axis

To characterize the movement of the display we tracked multiple parts of the lizard in successive frames using Matlab (Mathworks Inc). We tracked the position of the eye to characterize the push-up component (Fig. 2a), and multiple parts of the tail in each frame. We used the base and the tip of the tail and then chose three intermediate points that divided tails into equal-sized segments, with the constraint that the selected point was a clear and identifiable mark on the tail that facilitated tracking in all frames. As such, the relative position of the tracked points varied slightly between individuals. Following the procedures of Hedrick (2008), each point (eye and tail points) was located frame-by-frame from both camera views separately. The position data from both camera views and the calibration coefficients are then combined using direct linear transformation to represent the motion in 3D as xyz coordinates (Figs. 2a, 3b). For each point on the tail, we calculated the distance moved between successive frames (Fig. 3c) and then summed them over time to yield the total distance moved (Fig. 3d). The tail component of *A. muricatus* displays occurs predominantly at the distal part of the tail (Fig. 3e), non-uniformly across the five points. The distal parts of the tail exhibit steady incremental movements over time, and to a lesser extent so does the middle point. In contrast, the base of the tail and second point in most displays do not exhibit noticeable movement until later in the sequence. Based on this analysis, we selected the tip of the tail for use in subsequent analyses.

### Comparing signal amplitudes

**Fig. 4:**
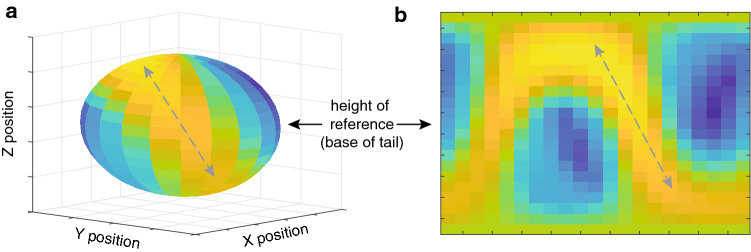
Visual angle generated by a push-up movement from multiple viewing positions around the animal. Relative angles are depicted as **a** a 3D sphere centred at the base of the tail, and **b** flattened into a 2D heat map. Larger amplitudes are shown in yellow and the grey arrows show corresponding data in each plot. The results suggest that perceived push-up amplitudes will vary with viewing angle

Our objective was to compare and contrast the amplitudes of push-up and tail-flick components. The general approach we followed was introduced by New and Peters (2010), and involves computing the angle subtended at a reference point (an intruder) for pairs of position data (Fig. 2b). We assumed an intruder could be positioned anywhere around the signaller, and used the *sphere* function in Matlab (Mathworks Inc) to generate equally spaced viewing positions around the lizard at a viewing distance of 1 m from the base of the tail (Fig. 4a). We used the default setting that creates 20 × 20 faces around the lizard and used the vertices of these as our position data for a total of 441 potential receiver positions. The two components differ substantially in duration. The push-up is rapid and completed in approximately 1 s, while tail flicking varies substantially (6–25 s in the present study but can continue for longer). We chose to partition tail flicking into non-overlapping segments of 1 s. For each push-up sequence and the 1 s tail flicking segments we computed the visual angle subtended at a given viewing position for all pairwise combinations of position data within the specified time window using the formula

$$ {\text{angle}} = a\tan 2\left( {{\text{norm}}\left( {{\text{cross}}\left( {v1,v2} \right),{\text{dot}}\left( {v1,v2} \right)} \right)} \right), $$

where *v*1 and *v*2 are separate lines connecting the viewing position and each of the two position data points, *a*tan2 computes the four-quadrant inverse tangent, and cross and dot are the cross-product and dot-product functions. We converted values from radians to degrees and selected the maximum angle in each case to represent the angular displacement of the movement from that viewing position. We repeated this for all viewing positions, resulting in 441 measures of angular displacement for each push-up or 1 s tail flicking segment. To facilitate inspection of these data, we present the 3-dimensional data in a flattened format (Fig. 4b). We examined the dynamic nature of tail-flicking and compare and contrast the maximum angular displacement achieved by push-up and tail flicking movements for our 8 display sequences.

## Results

**Fig. 5:**
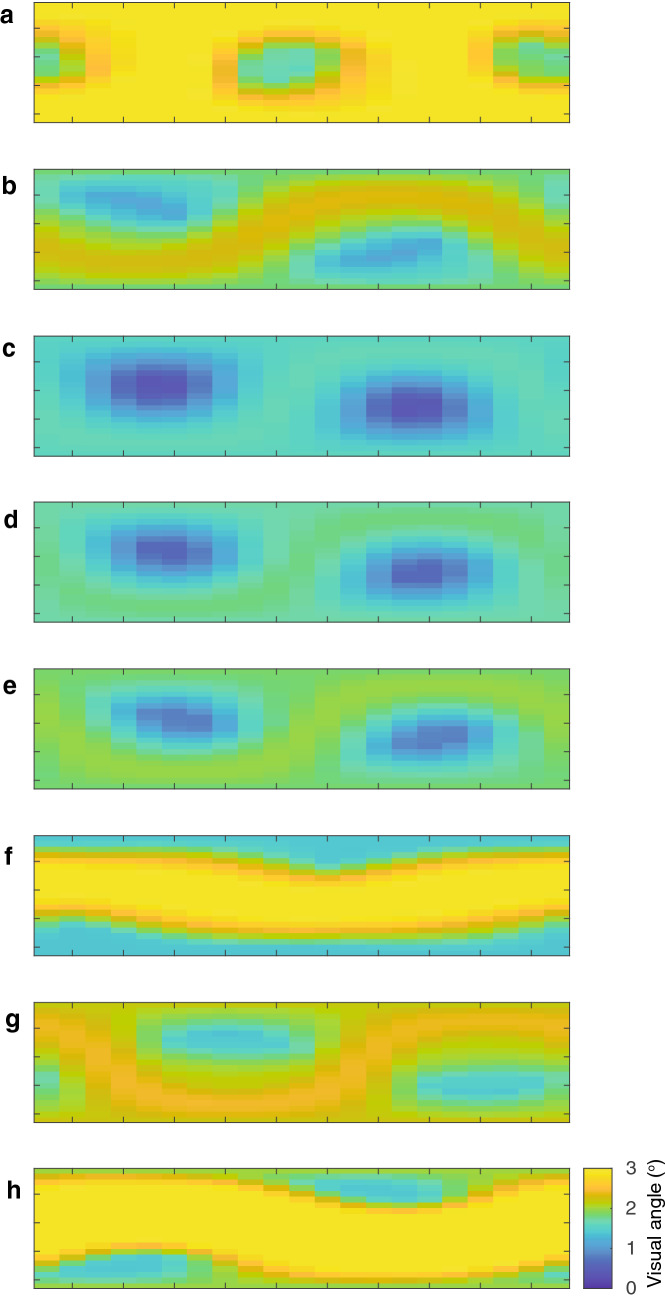
Heat maps depicting visual angle generated by push-up movements (~ 1 s) for eight displays (**a**–**h**) by *A. muricatus*. Values represent maximum visual angle for any pair of points during the push-up movement

**Fig. 6:**
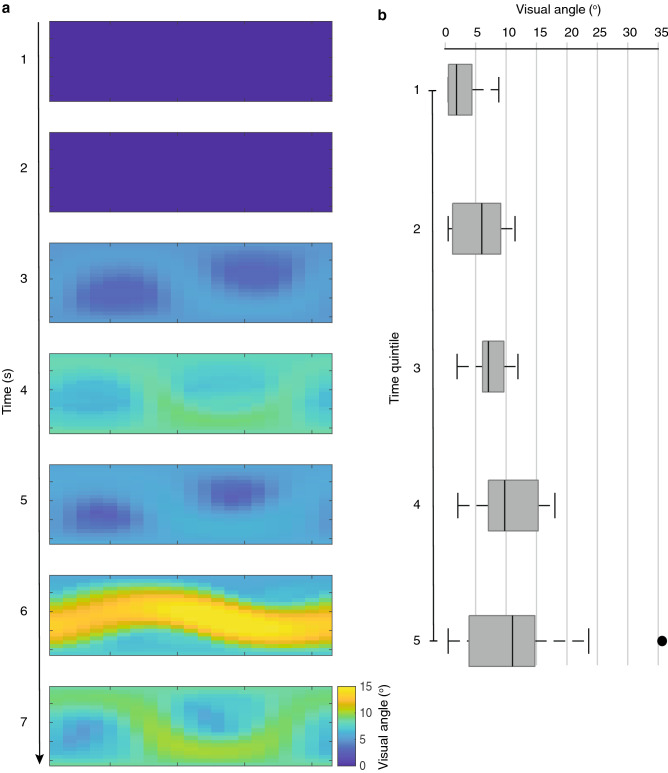
**a** Heat maps depicted visual angle generated by tail movements from a representative tail-flick sequence broken up into 1 s segments. **b** Box plot showing visual angles generated for all eight tail-flick sequences by *A. muricatus*, dividing each display into time quintiles. Tail movements start at relatively low amplitudes, with increasing amplitudes as the sequence progresses

**Fig. 7:**
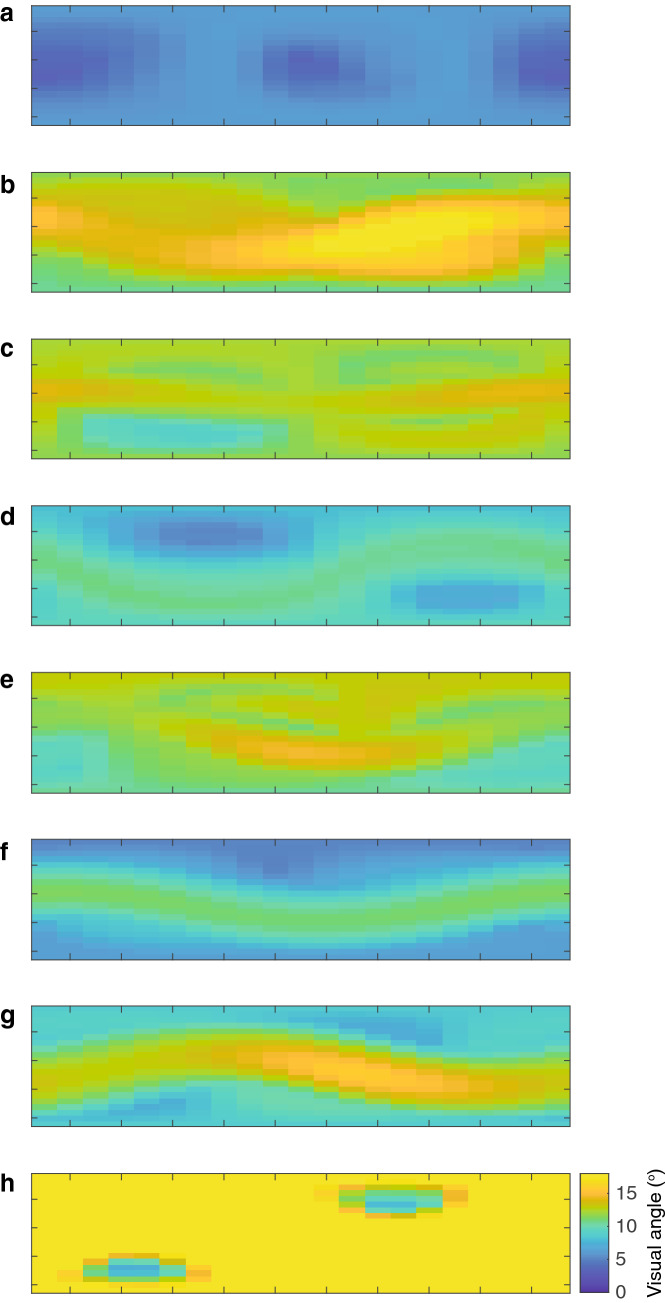
Heat maps depicting visual angle generated by tail-flicking movements for eight displays (**a**–**h**) by *A. muricatus*. Values represent maximum visual angle for any pair of points during the tail flicking sequence

**Fig. 8:**
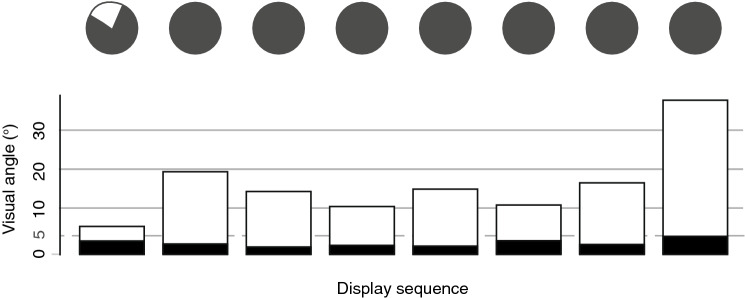
Bar chart representing the visual angles generated by push-ups and tail flicking for display sequences of eight *A. muricatus* lizards. Push-ups are shown as black bars and represent the maximum amplitude achieved from any viewing angle. Tail-flick amplitudes are shown as white bars and represent the maximum amplitude from any viewing angle within a 1 s time block. White segments within the pie charts above the bars reflect the relative proportion of viewing positions in which the maximum visual angle achieved by a push-up exceeded that of tail movements

Angular displacement of push-up movements is not uniform but varies as a function of viewing position (Figs. 4, 5). The sinusoidal pattern that characterizes all exemplars implies that optimal viewing positions are not defined by either the azimuth or elevation, but through their combination. The maximum angular displacement across all push-up sequences from any viewing position was 4.1°. Tail flicking shows notable variance over time (Fig. 6a) and also exhibited similar variance across viewing positions (Fig. 7). To characterize further the time-varying nature of angular displacement of the tail, we divided tail-flick sequences into five equally spaced time bins (quintiles) and allocated each of the 1 s segments from each display into one of these depending on the relative position of that segment within the overall duration of that sequence. Angular displacement tends to increase over time (Fig. 6b), with initial amplitudes typically less than 5° and steadily increasing over time to a median of more than 10°. Clearly, tail flicking has the capacity for large amplitudes (Fig. 7), with the maximum angular displacement across our 8 exemplars reaching 35.6^o^ (Fig. 6b). An angular displacement of this magnitude at a viewing distance of 1 m can be achieved only when the tail moves from a position of almost full extension in one direction to full extension in an opposite (180°) direction. In Fig. 8 we compare directly the maximum angular displacement of push-up movements and tail flicking. Here, the maximum angular displacement of tail flicks from any 1 s segment and from any viewpoint far exceeds the maximum value achieved by the push-up movement. Furthermore, every viewing position around the tail resulted in amplitudes greater than the maximum push-up value for seven of the eight exemplars.

## Discussion

Our analysis has demonstrated that the amplitudes generated by tail movements were consistently greater than that of push-ups over a comparable time window. Furthermore, the relative advantages of tail movements in this regard were apparent from all viewing positions around the signaller. Our approach simplifies the motion signals of both display components by characterizing movements at a single point. There are likely to be complexities of signal structure that we missed, but our method follows standard practice in quantifying lizard displays (Carpenter et al. 1970). While we are seeking alternative analytical approaches (see below), we suggest that our results demonstrate that the use of tail flicking at the start of displays reflects the ecological circumstances in which *A. muricatus* signals. It provides movement of high relative amplitude for receivers from any viewpoint and fits with playback studies that show detection is robust to orientation differences (Peters and Evans 2007). On the basis of movement amplitudes, tail movements can also be expected to be effective over a greater range of distances, and are a good option for dealing with motion noise within the environment. We outline our reasoning below.

**Fig. 9:**
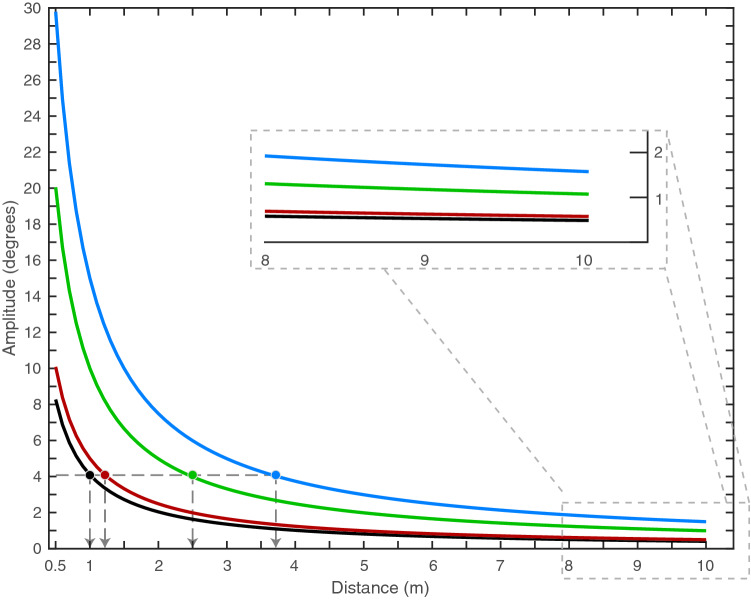
Relationship between movement amplitude and receiver distance for representative push-up (black line) and tail-flicking (coloured lines) movements. We selected the maximum amplitude of push-ups at a viewing distance of 1 m (4.1°), as well as relatively small (5°; red line), medium (10°; green line) and large (15°; blue line) tail movements. Small tail movements are typically found in the early part, while medium tail movements are found toward the latter half of the display. Large tail movements usually end the display, with the value we have chosen here only half of the maximum amplitude obtained when viewed at 1 m. Using the displacement values that generated these amplitudes at 1 m, we computed visual amplitudes over-viewing distances of 0.5–10 m. The circles and dashed arrows (lower-left) indicate amplitudes of 4.1° (an upper limit for push-ups at 1 m) and show the increased active space that can be achieved with tail movements of different magnitudes. Inset: enlarged portion of the plot showing that medium and large amplitude tail movements exceed 1° at 10 m. Large tail movements would not fall below 1° until after 15 m (data not shown)

Movement amplitude is a key parameter for movement-based signals of lizards (Fleishman 1986). On the basis of sensory system function, Fleishman and Pallus (2010) predicted that movement amplitudes influence the distance over which display movements can be detected. Movement amplitude was subsequently shown to be the parameter of head-bob displays modulated by *Anolis gundlachi* in response to varying receiver distances (Steinberg and Leal 2013), and the parameter that was adjusted by *A. sagrei* in response to changes in predation pressure (Steinberg et al. 2014). These adjustments serve to vary the active space of the signal and have been shown to vary in the movement-based displays of other taxa (How et al. 2008). However, Peters and Allen (2009) demonstrated that *A. muricatus* do not adjust tail displays in this way in response to receiver distances. Amplitude variability was a feature of each tail flicking sequence, but amplitudes were (almost) always greater than push-ups in this study. This might serve to allow signaling by *A. muricatus* over a greater range of distances. Our analysis held signaller-receiver distance to a constant of 1 m, but in Fig. 9 we examine the change in amplitude with viewing distance to show the active space advantages of different magnitudes of movement. Although the minimum angular size that can be detected by *A. muricatus* is not known, we expect it to be less than 1°. Tail-flicking by these accounts certainly has a much greater active space than push-up movements. However, signal exchanges will remain constrained by the distance over which other key components of the display (e.g., push-ups) can be detected, as well as the receivers ability to resolve the tail. Consequently, we do not see increased active space advantages to be the only reason why *A. muricatus* uses tail flicking as the introductory component.

Signaling strategies also reflect the environment in which they are generated (Endler 1992; Endler and Basolo 1998). The position of the animal relative to surrounding plants can influence the effectiveness of display movements (Peters 2010). It is hard to characterize in a simple way the amplitude of plant movements as there are typically multiple moving parts that are not independent of each other (Peters 2013). What is clear is that they can be distracting to receivers (Peters 2008). Adjusting movement amplitude is one strategy for overcoming the potential interference caused by plant movements. However, this will be more challenging when signaling close to plants as amplitude changes will need to be more substantial (Peters et al. 2008). Movements like push-ups are physically limited and our data show that they have a maximum amplitude much lower than tail movements. Tail movements are therefore preferable, although there is clearly more to the story as a single movement might achieve the desired amplitude yet tail flicking can be long lasting. We have computed amplitudes based on positions of the tip in 3D space but the tail is a thin structure that tapers from the base to the tip. This means that the part of the tail the generates the most movement, and the largest amplitudes might also be more difficult for the receiver to resolve against a cluttered background. As such, we suggest that not all *equivalent* amplitudes are equal. It is possible for this reason that tail flicking is performed over an extended period of time. Most of the sequences examined in the present study were 6–8 s duration, with one sequence lasting 25 s. In other work, the duration of tail-flicking in this species can extend beyond 120 s (Peters et al. 2007). It has been demonstrated previously that sustained movement is effective when the objective is to attract receiver attention but the movement need not be continuous (Peters and Evans 2003) and *A. muricatus* will lengthen the duration of tail flicking in response to increased levels of environmental noise (Peters et al. 2007). The lizards are not necessarily trying to ensure every movement is greater in amplitude than plant movement, but that they are likely to be generating enough over a period of time that are. The intermittent nature of the movement is also advantageous as the onset and offset of movement is highly salient to visual systems (Ibbotson and Clifford 2001). With this in mind, we suggest that the requirement to signal for longer is a function of the signaling environment, with the results of the present study suggesting that tail movements are ideally suited to serve this function. Extended signaling does have potential costs in terms of increased risk of predation (Koga et al. 1998). However, an advantage of using the tail in this way is that the head remains still, which will aid in the visual detection of predators, and the limbs are ready for the flight to cover if needed.

Different microhabitats generate distinct image motion environments (Peters et al. 2008). Although particularly challenging for motion-based visual signals, recent work has begun to quantify signal performance in different habitats (Ramos and Peters 2017; Bian et al. 2021). Translocating species between different habitats is not permitted (in Australia) so Bian et al. (2021) used sophisticated 3D animations to simulate different microhabitats and signaling species and found that the masking potential of densely vegetated environments, like the ones inhabited by *A. muricatus*, is greater than other habitat types used by Australian agamid lizards. We are just beginning this kind of work but it does seem likely that *A. muricatus* has developed a signaling strategy that is the product of the signaling context, including the environment in which they are typically found. The ecological challenges presented to them are not common for Australian agamid lizards and could explain the differences between species. Our knowledge of this group, and this mode of signalling, are limited by a lack of basic natural history knowledge (Ramos and Peters 2016), but we see no reason why the interaction of ecological context and sensory system function would not be relevant. The songs of birds (Slabbekoorn and Peet 2003; Slabbekoorn and Boer-Visser 2006) and the spectral properties of lizard dewlaps (Leal and Fleishman 2002) directly reflect ecological conditions, and we suggest that so do the movement-based displays of lizards.
